# Online learning during school closure due to COVID-19

**DOI:** 10.1007/s42973-021-00079-7

**Published:** 2021-06-10

**Authors:** Masato Ikeda, Shintaro Yamaguchi

**Affiliations:** Graduate School of Economics, University of Tokyo, 7-3-1 Hongo, Bunkyo-ku, Tokyo, Japan

**Keywords:** Education, COVID-19, Online learning, School closure, I24, I28

## Abstract

This paper estimates the effects of school closure on students’ study time and the number of messages sent from teachers to students using an online learning service. We find that both study time and message numbers increased significantly from the beginning of the school closure but they returned to pre-COVID-19 levels when the state of emergency ended in late May 2020. In addition, we find that students with prior access to the online learning service at home and students at higher-quality schools increased their study time more than other students. However, we find no gender differences in these outcomes.

## Introduction

At the peak of the educational disruption caused by COVID-19, over 1.5 billion students in roughly 190 countries missed out on learning at school.^1^ Given the scale and duration of school closures under COVID-19, the learning loss is likely to result in long-term negative consequences. During school closures, governments attempted to minimize the adverse effects. For instance, in at least 34 states in the US, local entities such as school districts and state departments of education partnered with public television stations to support online learning for students and teachers.^2^ The severity of the educational disruption, including whether government attempts to alleviate it were successful, has not yet been examined in detail.^3^ To contribute toward this gap in the literature, in this paper, we document how an online learning service enabled students to study during the COVID-19 pandemic in Japan.

The Japanese government began a nationwide school closure on March 2, 2020, which continued for 3 months until the end of May 2020. There is evidence that the school closure was unexpected and that many schools were unprepared for online learning. In this paper, we estimate the effect of school closure due to COVID-19 on the utilization of an online learning service by comparing user logs in 2020 with those from 2019. Our findings assist in understanding how an online learning service could make up the lost studying time during COVID-19.

For these analyses, we have access to the data on user logs of the online education service, SuRaLa, in Japan. Although the service offers variety of materials for students in wide range of grades, in this study, we focus on user logs of lecture materials and management functions for junior high school and high school students. Important feature of our dataset is that it includes students and their teacher who subscribed the service on a school basis. In other words, schools, rather than students and parents, made a final decision, upon introducing the service. This may imply that, conditional on being a student of a school in our sample, we do not expect household-level selection into adopting the service.

We find that the school closure during COVID-19 increased the study time of students using the online learning service, with the effect being strongest at the beginning of the school closure period. It decreased gradually and disappeared in June 2020, when most schools reopened. This result suggests that the online learning service enabled students to study online and compensated for the missed classes during the closure period. In addition, we find a positive effect on teachers’ effort levels, measured by messages sent from teachers to students via the online learning service. Interestingly, the effect on teachers’ effort is positively correlated with the effect on study time.

The contribution of this paper is to provide further evidence for the effects of online learning during the current COVID-19 pandemic. A few studies have investigated education under COVID-19, mostly with a focus on online learning (Bacher-Hicks et al., 2020; Chetty et al., 2020). Our study is most closely related to Chetty et al. (2020), in that both describe the utilization of an online education platform. Given that Figlio et al. (2013) find that online learning is a reasonable substitute for face-to-face learning, our results suggest that online learning services have mitigated the negative consequences of COVID-19 on education.

The remainder of the paper is organized as follows: In Sect. 2, we discuss the related literature. Section 3 outlines the institutional background and the online learning service. In Sect. 4, we describe our data and Sect. 5 presents and discusses our results. Section 6 offers concluding remarks.

## Related literature

Some studies based on observational data find negative effects of online lectures on learning (Bacolod et al., 2018; Bettinger et al., 2017; Xu & Jaggars, 2014). However, by comparing the estimates from observational and experimental data, Joyce et al. (2015) argue that the former are likely to overestimate the effect of face-to-face lectures. Figlio et al. (2013) show that an online lecture in a university increased students’ grade points by up to 3 points out of 100 relative to the effect of face-to-face lectures, although it had adverse effects for males and academically weaker students. Overall, these findings suggest that an online lecture can be a reasonable substitute for a face-to-face lecture for many students. Note that the result from the literature may not be directly applicable to junior high school and high school students because these studies examine university students.

Several studies investigate the impacts of COVID-19 on education. One strand of literature deals with real-time data under COVID-19. Chetty et al. (2020) examine data from an online learning service largely used as part of a math curriculum in the US. They observe an acute drop of the average number of lessons completed in the service during COVID-19, with a particularly strong negative effect for students in low-income areas. Aucejo et al. (2020) conduct a survey of 1500 students at Arizona State University, asking how COVID-19 affected their studies. They find a decrease in study time and a negative effect on educational outcomes, including delays in graduating and greater dropout from courses. Both Chetty et al. (2020) and Aucejo et al. (2020) describe how students’ study behavior is affected by COVID-19 and report that the total amount of study time decreased. Finally, Bacher-Hicks et al. (2020) use regional-level data on Google searches and find that the search intensity for online education material increased after the school closure and that the increase was more prominent in high-income regions.

We contribute to this strand of literature by documenting students’ and teachers’ activities in an online learning service during the COVID-19 pandemic. Our study is most closely related to Chetty et al. (2020). Similar to our study, they examine study behavior of K-12 students in an online education service, Zearn, under school closure during the COVID-19. They also conduct their analyses by plotting the transition of the number of lessons completed in the service before and after the COVID-19 outbreak. The major difference between our settings is that, prior to COVID-19, Zearn is used as a part of official curriculum in the US, while SuRala is primarily offered as supplementary educational materials. We suspect that this difference may account for why study time increased during school closure in our study, whereas Chetty et al. (2020) find a decrease in study time.

A second strand of literature examines the long-term effect of COVID-19 based on estimates from past incidents, such as the 2001 foot and mouth disease epidemic in England (Cook, 2020), and a combination of summer vacation, weather-related school closure, and absenteeism (Kuhfeld et al., 2020). Both Cook (2020) and Kuhfeld et al. (2020) predict a negative long-term effect of COVID-19. However, these studies may overestimate the effect of COVID-19 because, as Kuhfeld et al. (2020) state, online education can mitigate the negative effects.

## Background

In this section, we document the timeline of school closure due to COVID-19 in Japan to assist readers to understand the context. It should be noted that, in contrast to the situation in many other countries, the Japanese academic year begins in April and ends in March of the following year.

On February 27, 2020, Prime Minister Shinzo Abe made a public statement that the Japanese government requested all elementary schools, junior high schools, and high schools to close from March 2, 2020 until the beginning of spring break, which typically begins in the third or fourth week of March and lasts around two weeks, although the exact date and duration vary by region, school, and year. Thus, the statement implied roughly 3 weeks of school closure.

The announcement came as a complete surprise, as indicated by two pieces of evidence^4^ concerning the decision-making process of the government and the government school closure policy prior to the announcement. First, the Minister of Education, Culture, Sports, Science and Technology conceded in the Diet that Prime Minister Abe had only informed him of the school closure on the morning of February 27, the day of the public announcement. The Minister’s comment reveals that only a few people close to Prime Minister Abe were involved in the decision and that the information was not shared with the education minister, let alone with other policymakers, or teachers, parents, and students.

Second, prior to the announcement, the government’s school closure policy was, as requested by the Ministry of Education, Culture, Sports, Science and Technology, that each municipality’s school board should keep schools open unless a positive case of COVID-19 was found in a school. Given that most schools had no positive cases at that time, the school boards were unlikely to expect imminent school closures. In addition, on February 25, the Ministry requested that school boards begin making contingency plans for possible school closures.

The nationwide school closure due to COVID-19 began on March 2 and lasted until the end of May in most provinces.^5^ Following the government’s announcement, nearly all schools managed to close on March 2, only 4 days after the announcement. At that time, the government stated that schools would be closed for about 3 weeks, until the beginning of spring break. On March 20, the government announced that the school closure would not be extended after spring break and released guidelines for reopening schools on March 24. Hence, schools were expected to reopen after spring break, which was the beginning of the new academic year.

However, schools did not reopen after spring break because on April 7 the government declared a state of emergency for seven provinces, which was later extended nationwide. Along with this declaration, the government asked municipalities to decide whether to reopen schools at their own discretion. This declaration resulted in a de facto continuation of the school closure. As shown in Table 1, most schools remained closed, or reclosed, after the declaration of the state of emergency.

### Surala

Surala is an online learning service provider. Their service covers a variety of subjects, including math, Japanese, English, science, and social studies, and caters to all grades, from grade 1 to grade 12. Students can study materials above or below their own grade to review past materials or to prepare for university entrance exams. Subscription to the service can be on either an individual or a school basis. In this paper, we focus on users under school-wide contracts because this means that school and teacher characteristics are available. Students included in our data are enrolled in either junior high schools or high schools. Junior high school covers grades 7–9 and high school grades 10–12, but we omit grades 9 and 12 from our analysis, as discussed below.

The service provides three types of learning materials: lectures, drills, and tests. For the lecture material, students watch videos and answer quizzes during the lecture. As they can pause and rewind lectures, the time taken to complete a lecture varies between students. After each lecture, students are asked to solve drill questions. These differ from the quizzes during the lecture, which are primarily designed to draw students’ attention and ensure they understand the explanations provided in the lecture. By contrast, drills are problem sets designed to enhance students’ deeper understanding of the material.

A learning unit on Surala consists of a lecture and a drill. Several units relating to the same topic comprise one lesson. Finally, lessons are categorized into stages, depending on the level of advancement. Students may begin their study from any unit. Our data set includes user activity logs on units, but does not include any information on tests on Surala.

To manage students’ learning, Surala issues an account for teachers, which enables them to observe how their students study online. For instance, a teacher examines students’ understanding based on their progress and test scores with the learning service. In addition, teachers can send messages to students through the service, either to an individual student, a group of selected students, or to all students in the school.

## Data

### User activity log

We now describe our data set and define the variables used in the analysis. Our main data set is drawn from user activity logs on Surala. It includes information on when and what each student studied, and selected demographic characteristics, including grade and gender, as well as a school identifier. The data set also includes the teachers’ message log, which records the time and content of teachers’ messages to students.

#### Sample restrictions

We focus on students in junior high school (grades 7–9) and high school (grades 10–12), but we exclude the third-year students in both types of schools (i.e., those in grades 9 and 12) to avoid issues arising from attrition. Because the academic year ends in March and begins in April, most third-year students in both junior high school and high school move to a different school in April. Most junior high schools and high schools are separated, although some private schools provide a combined program.

We excluded observations for movers, students with missing information, and outliers. In the raw data, there are students who spent more than 10 h on one unit, whereas the average time to complete a unit is about 10 min. As we suspect that these students paid little attention to the material, despite being logged on to Surala, we exclude their entire study records from the data. Specifically, students with weekly study time exceeding the 99.9th percentile are dropped from the sample. Note that excluding outliers does not substantially change the mean study time, but slightly decreases the standard errors of our estimates. In addition, we excluded individuals with missing school identifiers for analyses that use school characteristics.

With regard to the message data, our sample includes messages sent from teachers to individual students. Although teachers can also send messages to a group of selected students or to all students in the school, in our data, 77% of messages were sent to individual students. In addition, when we construct a school-level variable, such as the weekly average number of messages per school, we include schools that used the service in January in our sample.

#### Summary statistics

Table 2 reports summary statistics for the key variables. The average time spent on completing a study unit is 8.98 min. During the period of analysis, 32% of students logged in to Surala in a given week. Among those who logged in, the average time for studying on Surala was 73.84 min. When we include students who did not log in (i.e., the majority), the average study time is 23.29 min. In our sample, 68% of students are in high school and 55% are males. Grades and gender were reported by the school as of April 2020.

We determine whether a student ever accessed the online learning service from home before school closure using the user log. Specifically, we consider that a student did not access the service from home prior to the school closure if he/she did not use the online learning service either after 8:00 p.m. or on the weekend during the period from April to December 2019. According to this definition, 17% of students had no prior access to the online learning service from home.

Our data include 224 schools, of which 136 agreed to disclose their names. They consist of 41 junior high schools, 85 high schools, and 10 combined junior and senior high schools. For these schools, we used a measure of school quality published by a private firm. The measured quality was originally scaled to a mean of 50 and a standard deviation of 10 among the schools on their list.^6^ For junior high schools, the list includes only selective private schools, whereas for high schools, both public and private schools are included. The average quality index for junior high schools in our sample is 40.30, whereas that for the high schools is 49.81.

There were 4,596 messages sent from teachers to students and 95% of them were sent in 2020. Among the schools from which at least one message was sent from a teacher in a given week, the average weekly number of messages sent from a school is 10.66 and the average weekly number of teachers who sent a message is 1.41. Among teachers who sent at least one message in a given week, the average weekly number of messages is 7.56.

**Fig. 1:**
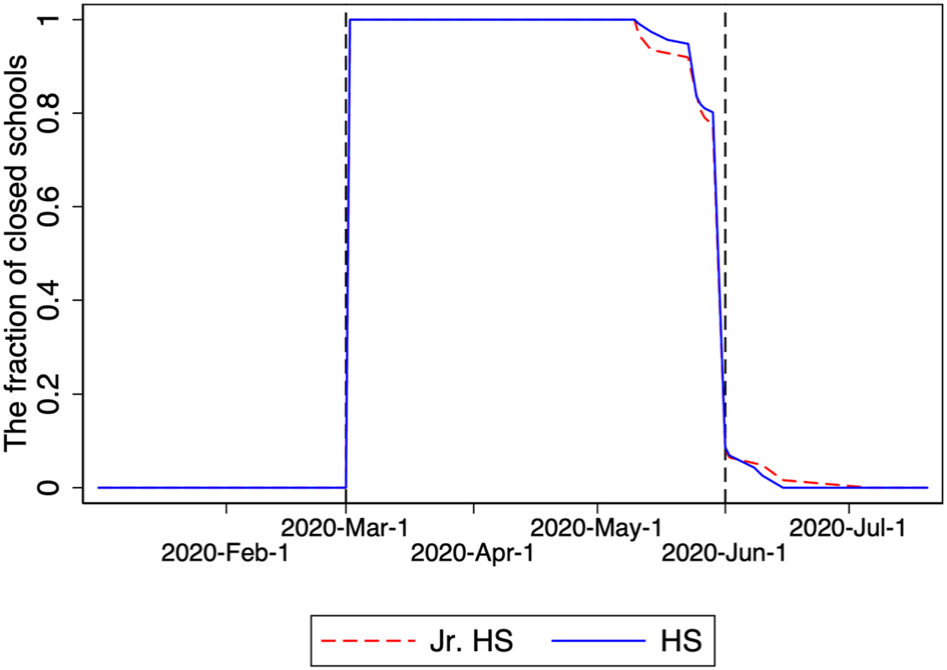
Fraction of closed schools in the sample. Jr. HS and HS denote the fractions of closed junior high schools and high schools, respectively

Finally, we collect data on the date of school closure. As described in Sect. 3, schools gradually reopened as the state of emergency was lifted in selected provinces on May 14 and in all provinces on May 25. Figure 1 shows the percentage of schools in our data that were closed. Most of them closed on March 2 and reopened from June 1, and this pattern is common across junior high schools and high schools.

## Results

### Students’ study time

**Fig. 2:**
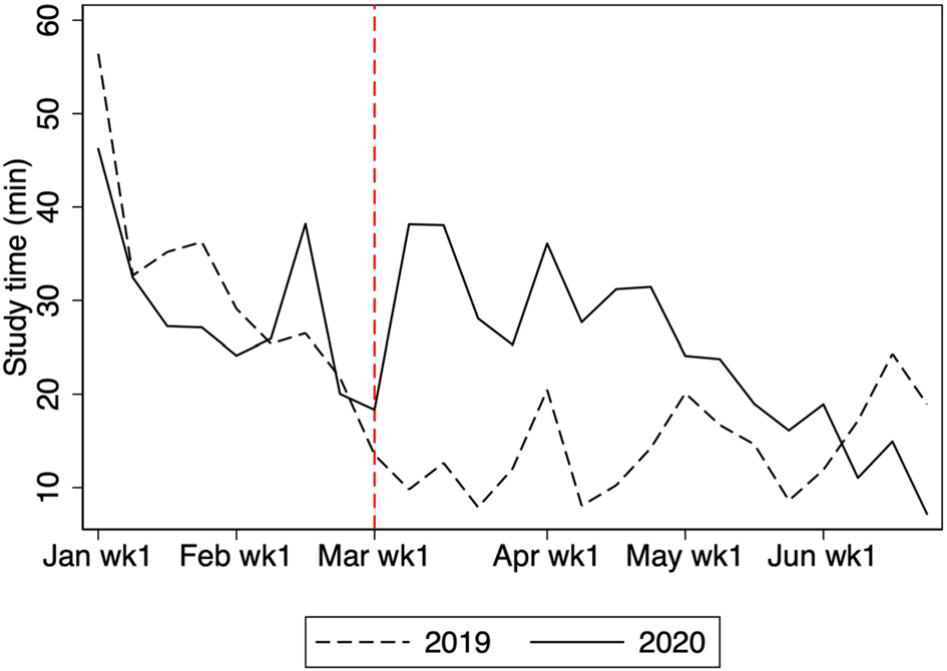
Average weekly study time. The figure shows the average study time for students in grades 7, 8, 10, and 11

In this section, we show how students’ study time and teachers’ messaging changed in response to the COVID-19 school closure. Figure 2 presents the average weekly study time in 2019 and 2020. We use study time in 2019 as a comparison to indicate what would have happened in 2020 if the COVID-19 pandemic had not occurred. The vertical dashed line (in red) indicates the beginning of the nationwide school closure. For the first week of January 2020 to the last week of February, there is little difference in study time between 2019 and 2020. However, the online study time surges in the first week of March 2020 relative to the same week in 2019. The average study time in 2020 continues to be longer than that of 2019 until the first week of June, when most schools reopened.

**Fig. 3:**
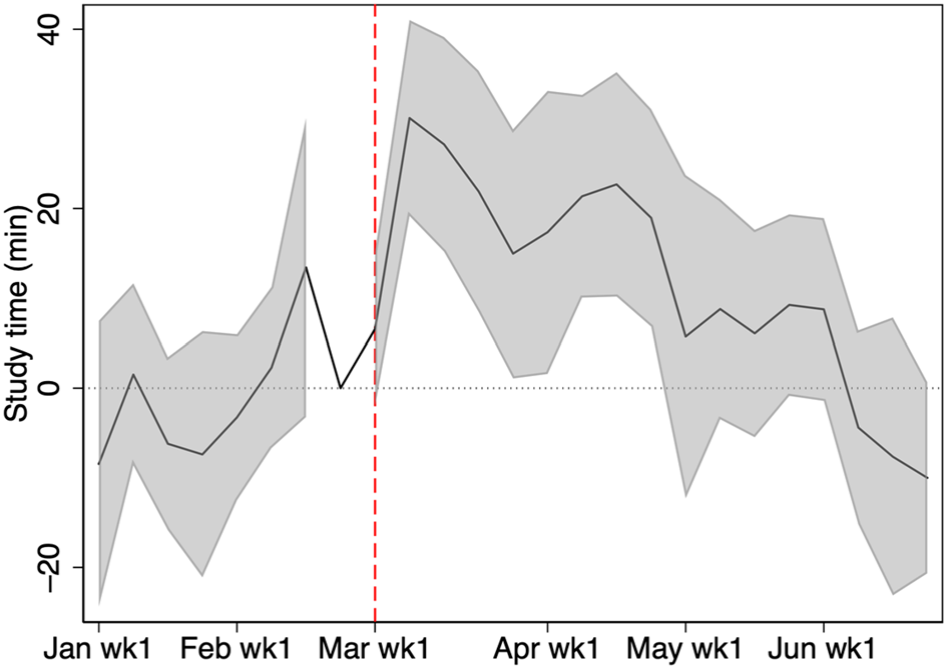
Change in weekly study time from 2019 to 2020. The shaded region is the 95% confidence interval computed with standard errors robust to clustering at school level. Study time is shown for students in grades 7, 8, 10, and 11

Figure 3 shows the growth of the average weekly study time from 2019 to 2020 with a 95% confidence interval. From January 1 until the end of February, there is no statistically significant difference. However, from the start of the school closure period, the study time in 2020 is significantly longer than the corresponding time in 2019. In fact, based on Table 3, the study time in 2020 is 22 min longer per week, which roughly amounts up to two 45-min-classes in one month. We observe a statistically significant growth in study time until the end of April. Although there is a growth in study time until the first week of June, the growth is statistically insignificant in May.

**Fig. 4:**
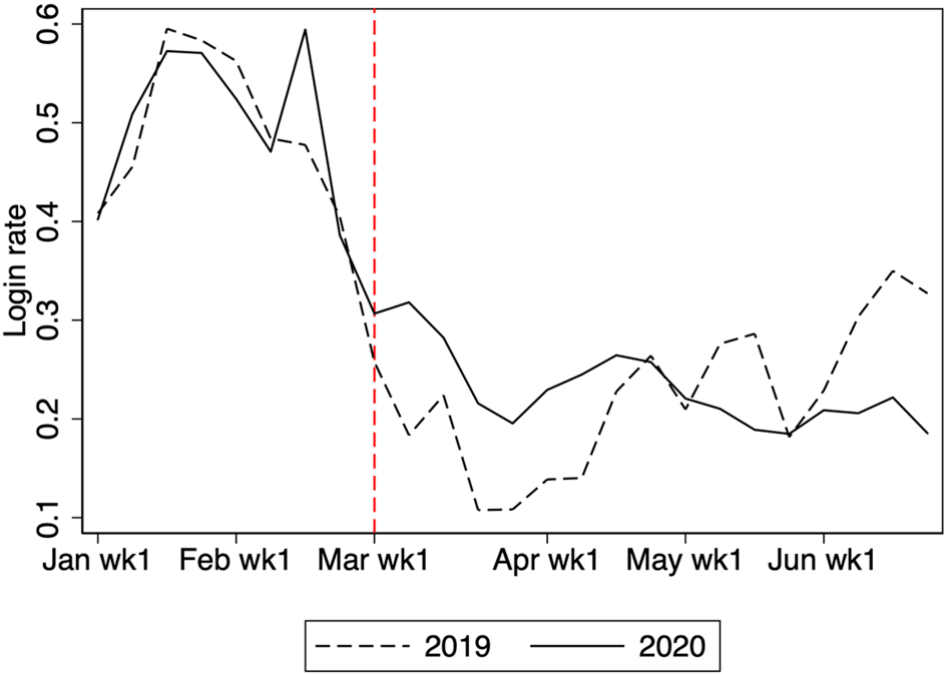
Weekly log-in rate. The figure shows the log-in rate for students in grades 7, 8, 10, and 11. Log-in is defined as studying at least once in a given week

**Fig. 5:**
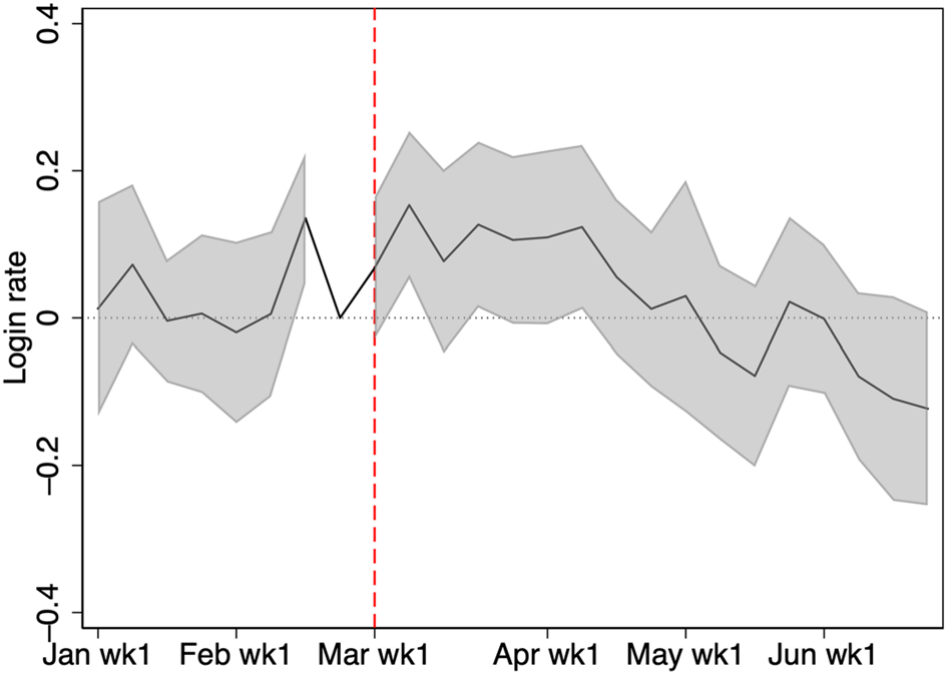
Change of weekly log-in rate from 2019 to 2020. The shaded region is the 95% confidence interval computed with standard errors robust to clustering at school level. The figure shows the log-in rate for students in grades 7, 8, 10, and 11. Log-in is defined as studying at least once in a given week

According to the weekly log-in rate in Table 2, only 32% of students study online each week. We expect that a large portion of students remain unaffected by school closure because they do not have access to the online learning service at home. Next, to determine the main driver of the overall effect, we examine the extensive and intensive margins of changes in study time. Figures 4 and 5 show changes in the extensive margin, defined by a weekly log-in indicator. They show that there is a marginally significant effect of school closure from March to the beginning of April. That is, the effect on the extensive margin disappears slightly earlier than the overall effect.

**Fig. 6:**
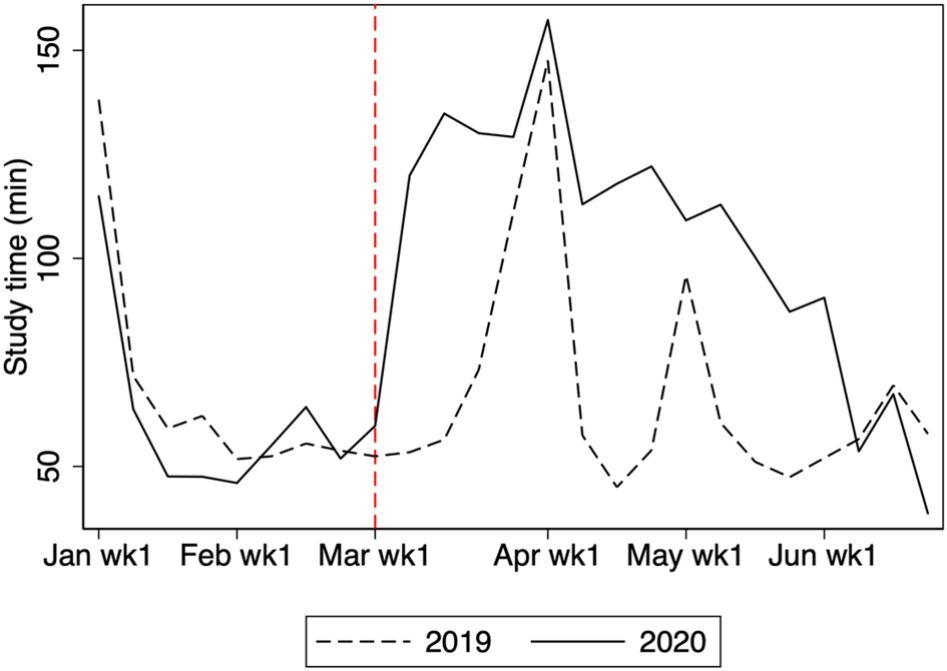
Average weekly study time conditional on log-in. Average study time conditional on log-in for students in grades 7, 8, 10, and 11. Only students who study at least once in a given week are included

**Fig. 7:**
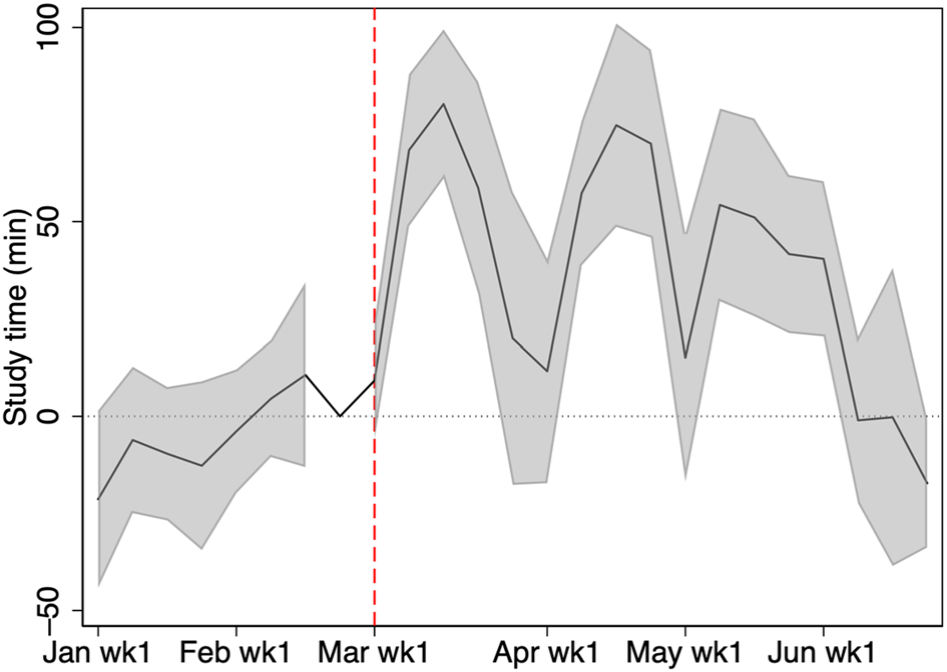
Change of weekly study time conditional on log-in from 2019 to 2020. The shaded region is the 95% confidence interval computed with standard errors robust to clustering at school level. The figure shows the average study time conditional on log-in for students in grades 7, 8, 10, and 11. Only students who study at least once in a given week are included

Figures 6 and 7 show the changes in the intensive margin, defined by the average weekly study time conditional on log-in. In 2019, there are spikes at the end of both March and April. These periods correspond to the end of spring break and the 1-week-long national holiday (Golden Week), respectively. A Surala manager explained the spikes by the fact that students study intensively to finish homework due after the spring break and the holidays. Regardless of the spikes, a significant positive effect for the intensive margin persists until the end of May. That is, the effect for the intensive margin persists longer than the overall effect. In fact, monthly analyses in Table 3 also shows that the magnitude of the effect on intensive margin is almost as large as the previous month while the effect on overall effect decreases by half in May.

Finally, Table 3 summarizes results from analyses of monthly log-in rate and study time, which shows patterns consistent with the weekly analyses. Note that, in these analyses, study time is defined by weekly average study time in a given month. In Table 3, study time, as well as the intensive and extensive margin, responses to school closure in March, and the difference continues to be present by the time of schools reopening, June. For instance, the effect on unconditional study time in March is more than three times longer comparing to the effect in May. One difference is that we detect the statistically significant effect on unconditional study time in May. Overall, the analyses in Table 3 provide similar results to weekly analyses, with some improvement in precision.

### Teachers’ messaging

**Fig. 8:**
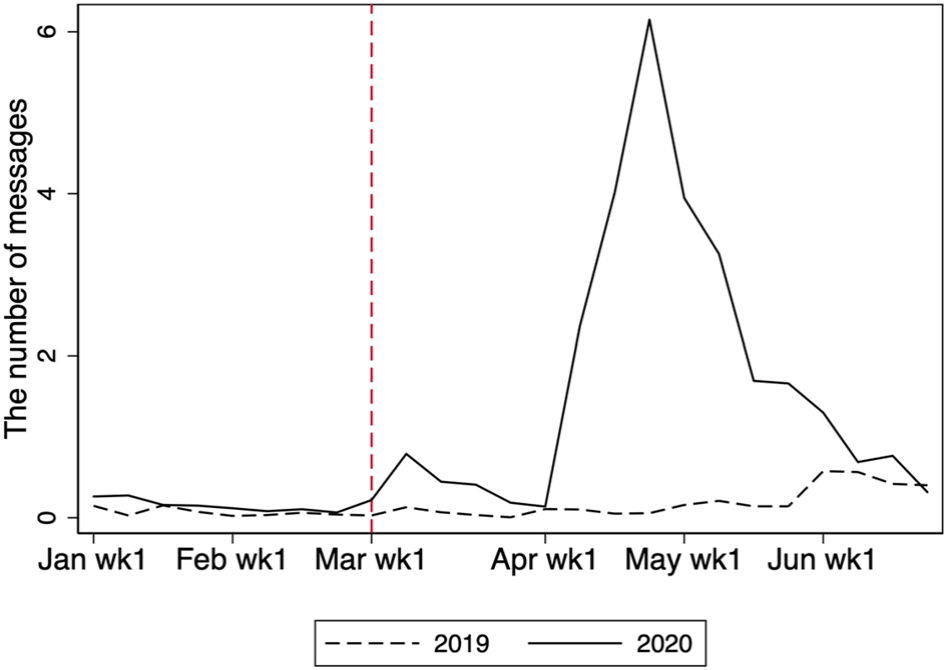
Total number of messages from teachers per week. The figure shows the weekly average number of messages from teachers to students per school

Not only students but also teachers responded to the school closure. Figure 8 shows the total number of messages sent from school each week. Before the school closure, teachers sent virtually no messages, presumably because they communicated with their students in person. The number of messages after school closure increased in the months of March, April, and May. The largest increase occurred in the middle of April, whereas study time was longest in March. In fact, as in Tables 3 and 4, the effect on overall study time in March is roughly three times larger than that in May, whereas the effect on message is five times less. As March is the end of the school year, students may not have required much support from teachers, given that they mostly review materials taught in class at this time. By contrast, in April, at the beginning of the new school year, students studying new materials may require more assistance from teachers, leading them to send more messages in April.

**Fig. 9:**
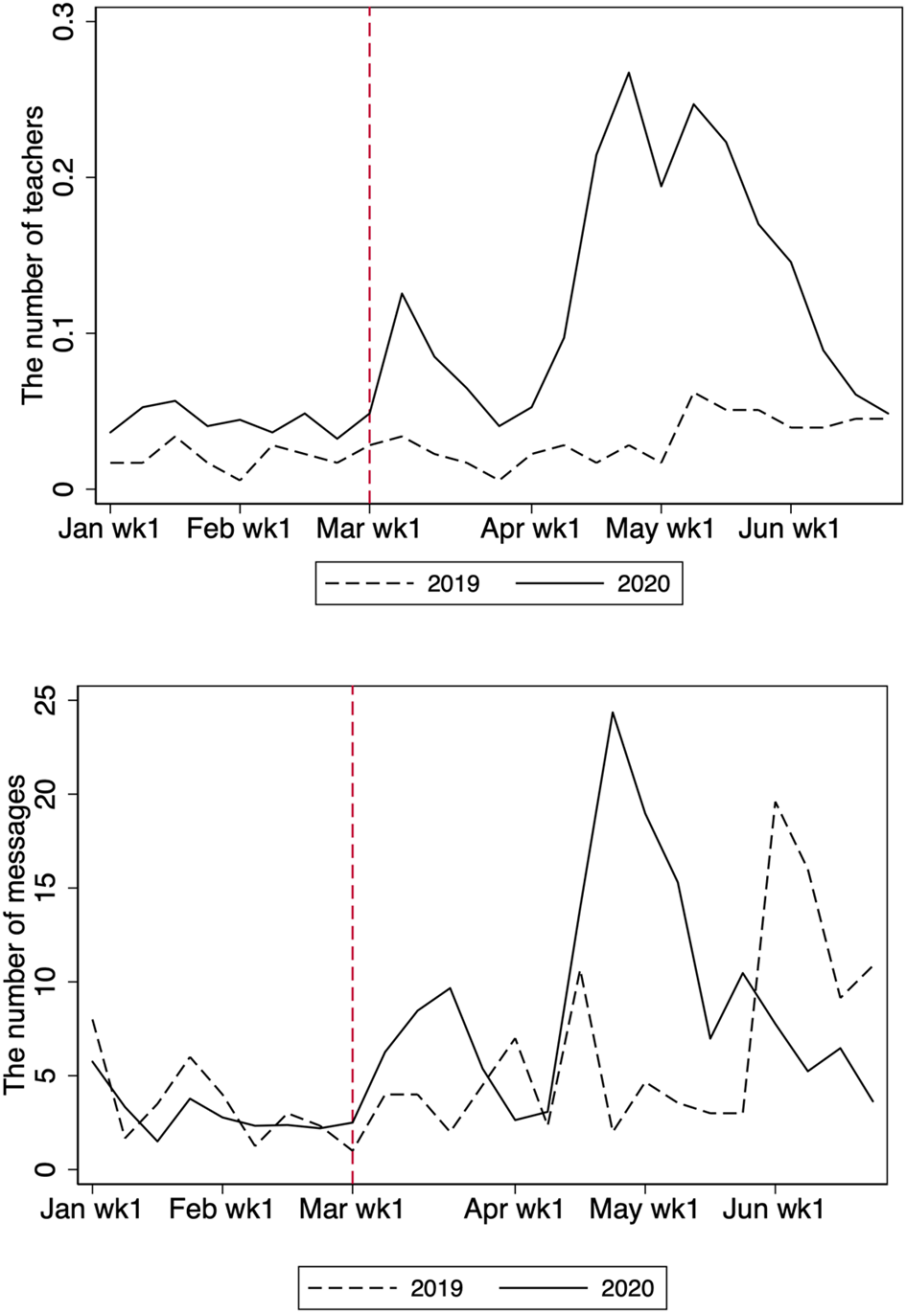
Numbers of teachers online (top) and messages sent per teacher. Teachers are defined as being online if they send at least one message in a given week. The number of messages per teacher is defined as the average number of messages per teacher conditional on being online

Figure 9 presents the number of teachers online and the number of messages sent per teacher. The upper panel of Fig. 9 shows the number of teachers who sent at least one message each week. While there were more teachers online in 2020 in any week, the movements in the number of teachers online are parallel between 2019 and 2020 for January and February. There was a rise in the number of teachers online in the second week of March 2020, with a further rise in the second week of April when the new academic year began. However, the number of teachers online fell to the pre-COVID-19 level in the second week of June 2020. The lower panel of Fig. 9 shows the average number of messages per active teacher online. This number moves in a similar fashion to the number of active teachers online, although the pattern is clearer in the upper panel. The magnitude of the change is largest in April, which is consistent with the effect on the total number of messages shown in Fig. 8. Overall, therefore, the changes in the aggregate numbers of messages were driven by both extensive and intensive margins.

### Correlation between study time and messages

Next, we examine the relationship between students’ study time and messages from teachers. One might expect that teachers paying attention to students by sending messages to them would encourage students to study more. Although we cannot test this hypothesis, we can examine the association between students’ study time and teachers’ messaging. Note that we exclude outliers, which we define as changes in study time exceeding 146 min (95th percentile) or changes in number of messages of more than 1337 (99th percentile). The results including the outliers are reported in Appendix IV.

**Fig. 10:**
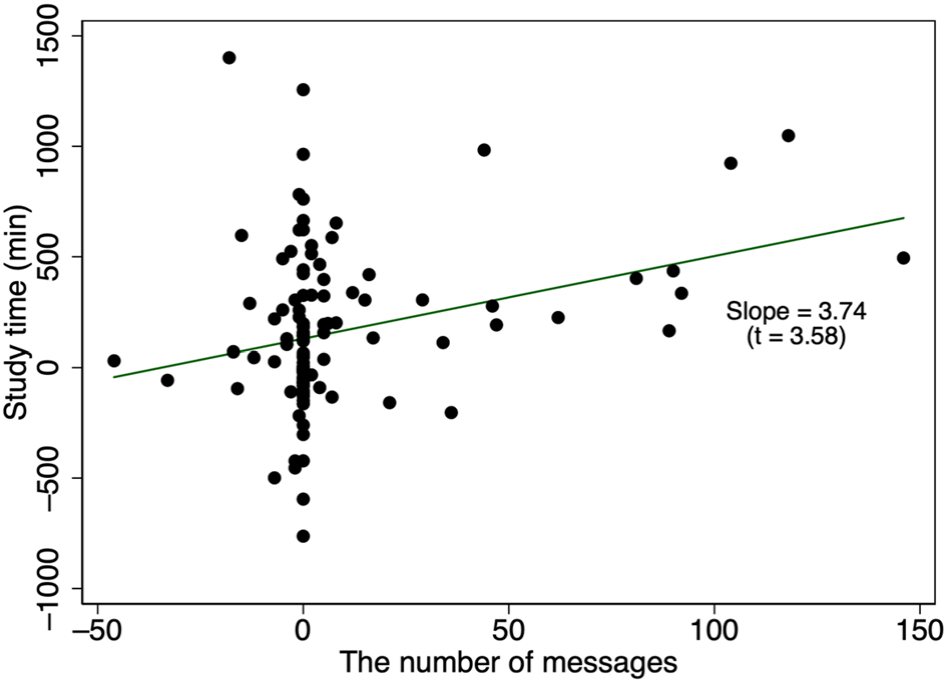
Correlation between study time and messages. Observations are excluded if the change in study time is greater than 146 min (the 95th percentile) or if the change in the number of messages exceeds 1,337 (the 99th percentile). The graph with the full sample is provided in Appendix IV. The number of observations is 123 and the unit of observation is the school. Study time represents the average study time within each school in March, April, and May 2020. The number of messages represents the total number of messages sent from teachers to students in the same period

As Fig. 10 shows, we find a positive correlation between changes in the school-level average study time and changes in the number of messages at the school level. The former is calculated by taking the difference in school-level average study time from March to May in 2019 and that in 2020, noting that school level means the average study time per student in each school. Similarly, a change in the number of messages at the school level is measured as the difference between the total number of messages from March to May in 2019 and that in 2020 in each school.

**Fig. 11:**
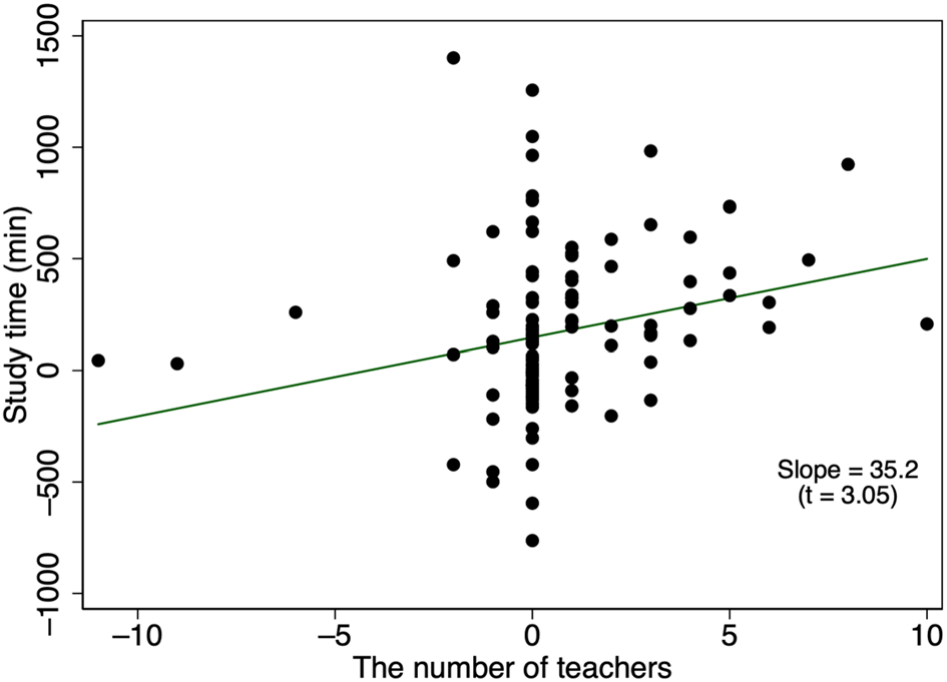
Correlation between study time and the number of teachers online. Observations are excluded if they involve a change in study time that exceeds 1,891.319 (99th percentile) or a change in the number of messages that is greater than 11 (the 99th percentile). A graph based on the full sample is provided in Appendix IV. The number of observations is 123 and the unit of observation is the school. Study time represents the average study time within each school in March, April, and May. The number of teachers online represents the number of teachers who sent a message at least once in March, April, and May

In Fig. 11, we observe a positive correlation between changes in study time and changes in the number of teachers online at the school level. We define a teacher being online as a teacher who sent at least one message during the period from March to May in 2019 or during 2020. School level means that we consider the number of teachers online in each school.

### Heterogeneity

In this section, we examine the heterogeneous effects of COVID-19 on study time by prior access to the online services from home, school quality, and student gender. In Appendix V, we report the heterogeneous effects by grade, region, past utilization, and in-class utilization. The analysis compares study time in 2020 between two groups in each case. For instance, we describe heterogeneity by school quality by comparing the weekly average study time of a high-quality school in 2020 with that of a low-quality school in 2020.

#### By prior access from home and school quality

Chetty et al. (2020) find that the number of lessons completed by students from different income areas varies during school closure. We suspect that this difference arises from variations in internet access at home and study habits. In the following, we examine heterogeneity in prior access to the online study services from home and school quality.

**Fig. 12:**
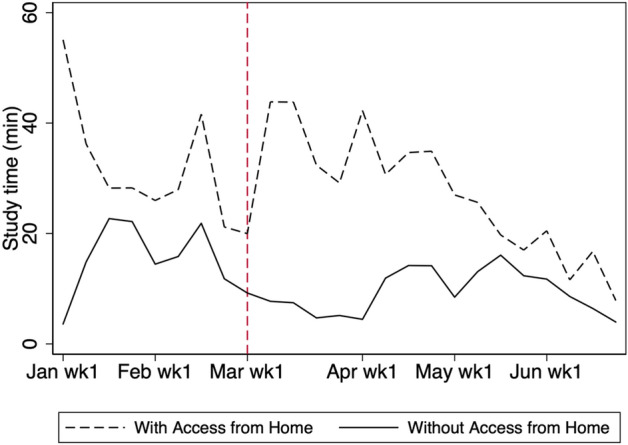
Average weekly study time by prior online learning access from home in 2020. The figure shows the study time by prior access to online learning from home for students in grades 7, 8, 10, and 11. Studying at home is defined by accessing the online services after 8:00 p.m. or on the weekend. A student who never studied at home from April to December in 2019 is defined as one with no prior access from home. Note that students who never used the service from April to December in 2019 are excluded

**Fig. 13:**
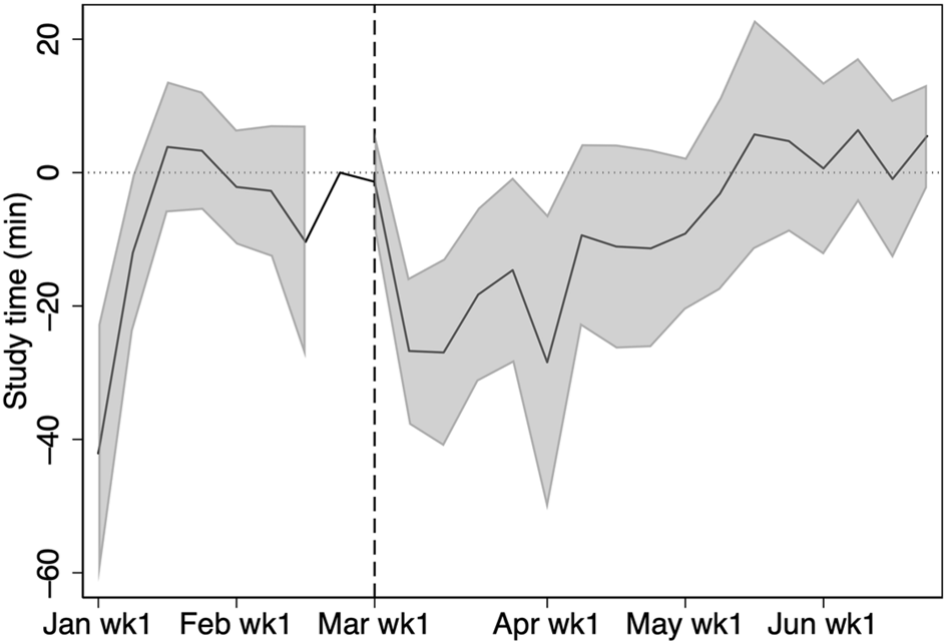
Average weekly study time by prior access from home in 2020. The shaded region is the 95% confidence interval computed with standard errors robust to clustering at school level. The figure shows the study time for students in grades 7, 8, 10, and 11 with no prior access to online learning from home minus the study time for students in grades 7, 8, 10, and 11 with access from home. Studying at home is defined by accessing the online services after 8:00 p.m. or on the weekend. A student who never studied at home from April to December in 2019 is defined as one with no prior access from home. Note that students who never used the service from April to December in 2019 are excluded

In Fig. 12, we compare the study time of students with and without prior access to online services from home. We consider that a student has no prior access to online services from home if he/she never logged in after 8:00 p.m. on weekdays or at any time on the weekend from April 2019 to the end of December 2019. Although the study time of students with prior access to online services from home increased after the school closure, the study time of those with no prior access decreased. As shown in Fig. 13, the difference decreased over time and continued to be statistically significant until the beginning of April.

We do not consider the large difference between the two groups at the beginning of January to be problematic for our identification because it is likely to have arisen from the company, SuRaLa Net Co., Ltd., promoting its services and the New Year holiday. The company’s promotion involved the students participating in a tournament, in which they were ranked by study times. Because students who could use the service from home would have found it easier to increase their study time in response to this promotion, they were likely to use the service more than students without access. In addition, as the New Year holiday occurs during the first week of January, students could not access the online service through school facilities at this time. This may explain why students with no prior access from home tend to study less at the beginning of January. However, except for this period, the trend in study time before the school closure period is similar between the two groups.

In summary, we observe that students with prior access to online services from home utilized the service more under COVID-19 than did students with no prior access from home.

**Fig. 14:**
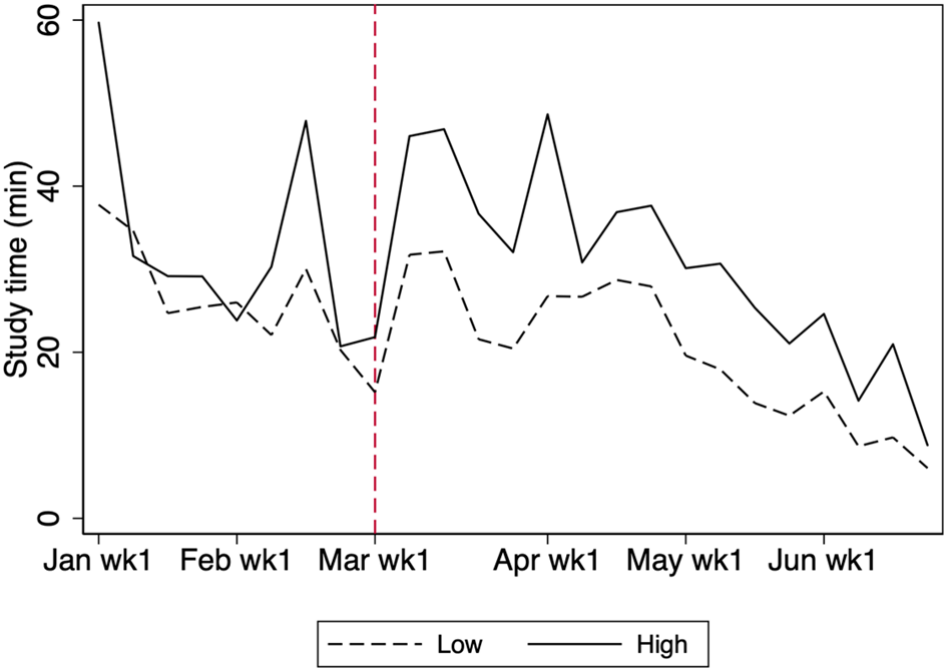
Average weekly study time by school quality in 2020. The figure shows the study time by school quality for students in grades 7, 8, 10, and 11. School quality is defined by the level of the school, which is obtained from external sources. Low and high describe schools for which the quality level is below and above the median, respectively

**Fig. 15:**
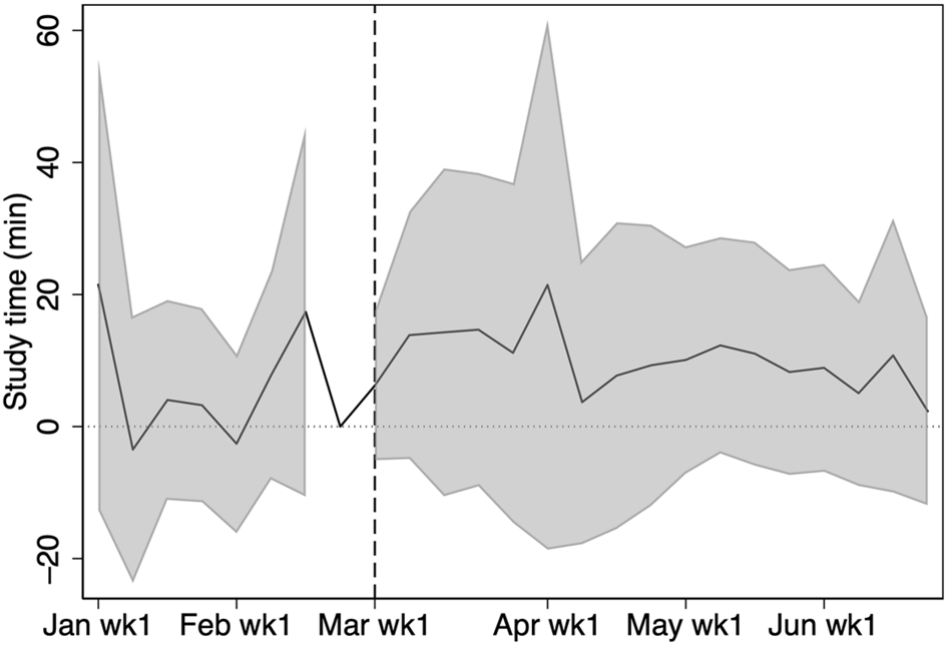
Heterogeneity by school quality. The shaded region is the 95% confidence interval computed with standard errors robust to clustering at school level. The figure shows the study time for students in grades 7, 8, 10, and 11 in high-quality schools minus the study time for students in grades 7, 8, 10, and 11 in low-quality schools. School quality is defined by the level of school, which is obtained from external sources. Low and high describe schools for which the quality level is below and above the median, respectively

Second, we examine students by the quality of their schools. We consider a school to be high quality if its quality index is above the median. Figures 14 and 15 describe heterogeneity with respect to school quality. We find that students from higher-quality schools consistently studied more during the school closure period, although the difference is not statistically significant.

#### By gender

**Fig. 16:**
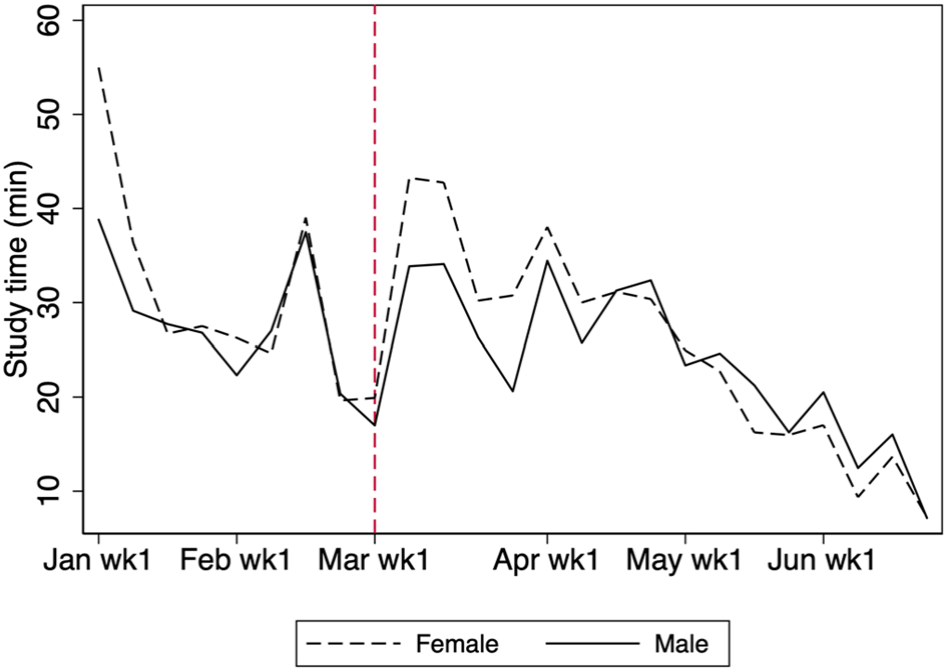
Average weekly study time by gender in 2020. The figure shows the study time by gender for students in grades 7, 8, 10, and 11

**Fig. 17:**
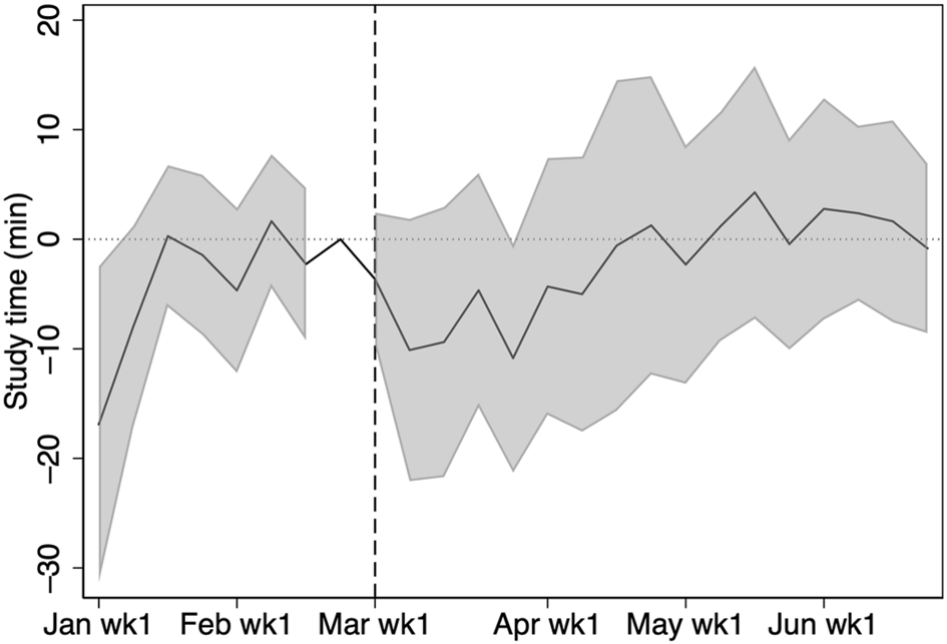
Heterogeneity by gender. The shaded region is the 95% confidence interval computed with standard errors robust to clustering at school level. The figure shows the study time for male students in grades 7, 8, 10, and 11 minus the study time for female students in grades 7, 8, 10, and 11

Figlio et al. (2013) show that male students tend to struggle with online learning; therefore, we examine any differences in study time between male and female students. Figures 16 and 17 show that there is no statistically significant difference in the average weekly study time between male and female students in 2020.

We also examine heterogeneity across several other variables, namely grade, region, previous usage, and in-class utilization. For most of these, we find no sizable difference across the two groups; however, the one exception is heterogeneity by grade. In fact, we find a statistically significant difference between high school and junior high school students. In addition, the result concerning heterogeneity by past utilization of the online learning service suggests that the schools with more experience of the service are more likely to use it both before and after the school closure period in 2020. More details of these results can be found in Appendix V.

## Summary and concluding remarks

This paper documents the effect of the school closure under COVID-19 on students’ study time and teachers’ inputs in an online learning service. We find that online study time significantly increased during the school closure and that it returned to the pre-COVID-19 level when the school closure lifted at the end of May 2020. In addition, we find that teachers sent more messages to students via the online service during the school closure than before or after. We note that the effects of school closure are heterogeneous. Specifically, students with access to the online learning service from home and students at higher-quality schools increased their study time more than other students.

Our finding suggests that an online learning service may help students to study during school closure. Thus, the government may want to consider introducing online learning tools in preparation for future possible school closures. Further, we note that policy makers should be aware that a lack of internet and/or personal computer access can raise inequality in learning during school closure.

A potential limitation of our study is that we do not have data on students’ learning activities outside the service. For instance, students who studied intensively offline would not have suffered learning losses under COVID-19 school closures, despite not studying via the online learning service. In addition, home/housing environment and home-study support by parents/siblings may affect students’ learning, but we are unable to observe them in the current data. Future research on the impact of COVID-19 on education should complement our results by examining a more comprehensive measure of the impact, such as long-term educational attainments.

## Figures and Tables

**Fig. 18:**
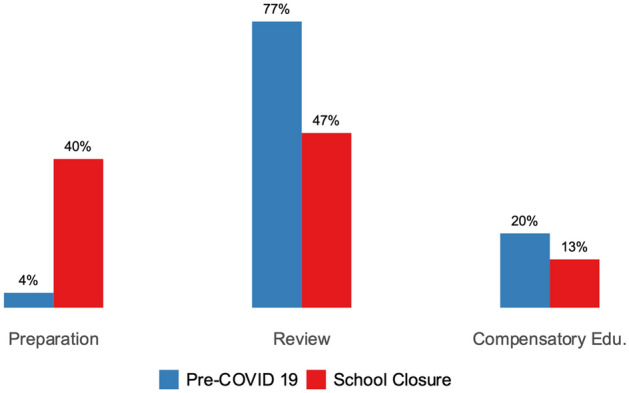
Purpose of using surala. This graph is created based on the information obtained from the interview to each school. The number of observations for Pre-COVID 19 and School Closure are 188 and 156, respectively. Preparation means the platform is used to let students study materials covered in class before they take classes. Compensatory Edu. Means the platform is used to study topics covered in class long time ago

**Fig. 19:**
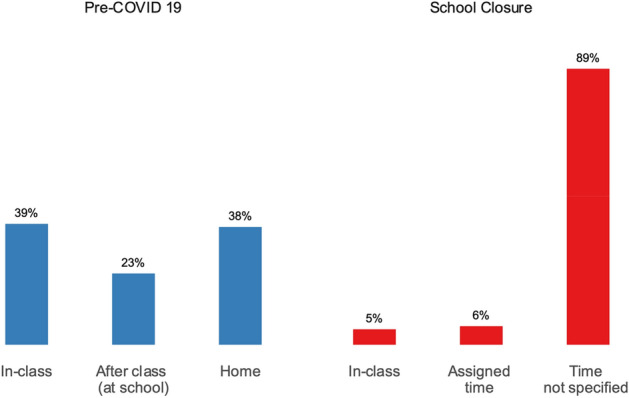
Occasion where schools use Surala. This graph is created based on the information obtained from the interview to each school. The number of observations for Pre-COVID 19 and School Closure are 188 and 163, respectively. Home means the platform is used homework. Assigned time means teachers tell students when to study in the platform. Time not specified means students are free to choose when to study in the platform

**Fig. 20:**
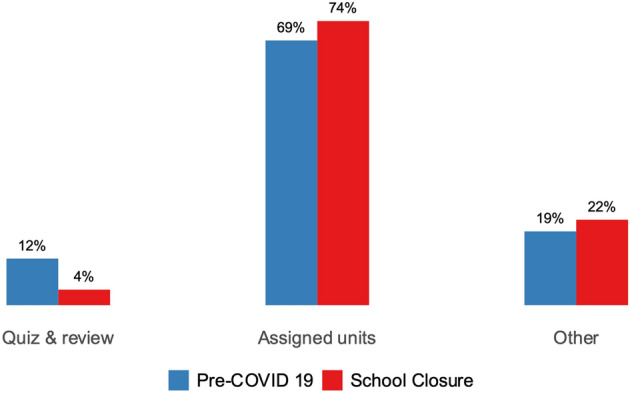
How to use Surala. This graph is created based on the information obtained from the interview to each school. The number of observations for Pre-COVID 19 and school closure are 188 and 163, respectively. Quiz and review means students first take quiz and study only the part they are not familiar with. Assigned units means teachers assign certain units to students

**Fig. 21:**
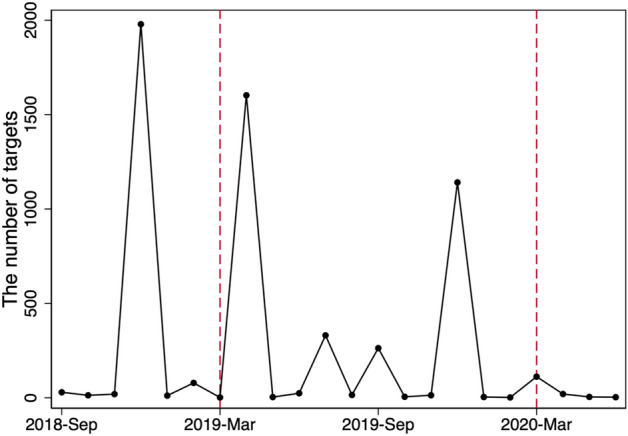
Weekly average number of targets based on set date. The unit of observation is school. The date of target is defined by the day when the target is set by a teacher

**Fig. 22:**
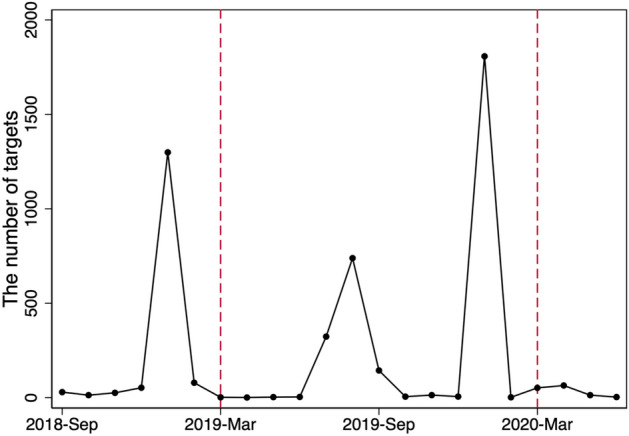
Weekly average number of targets based on end date. The unit of observation is school. The date of target is defined by the day when the target is supposed to be completed

**Fig. 23:**
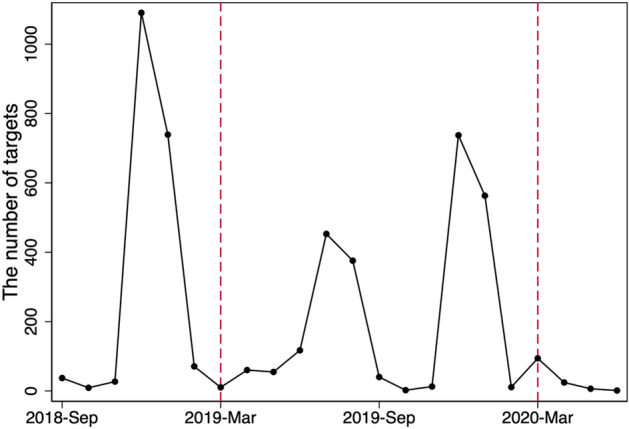
Weekly average number of targets based on complete date. The unit of observation is school. The date of target is defined by the day when a student finishes the target set by a teacher. We exclude students who did not complete the assigned target

**Fig. 24:**
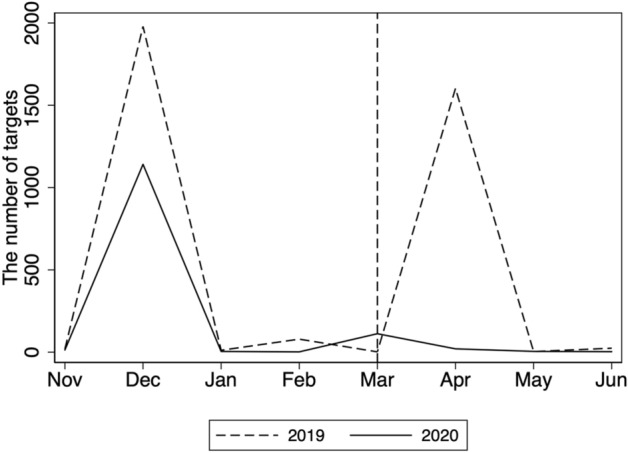
Difference in weekly average number of targets based on set date. The unit of observation is school. The date of target is defined by the day when the target is set by a teacher

**Fig. 25:**
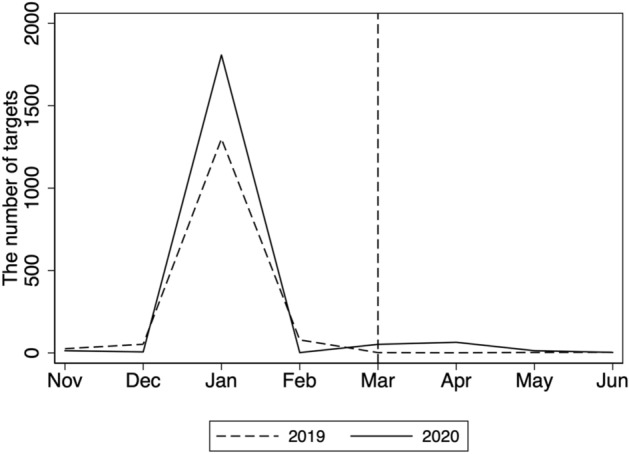
Difference in weekly average number of targets based on end date. The unit of observation is school. The date of target is defined by the day when the target is supposed to be completed

**Fig. 26:**
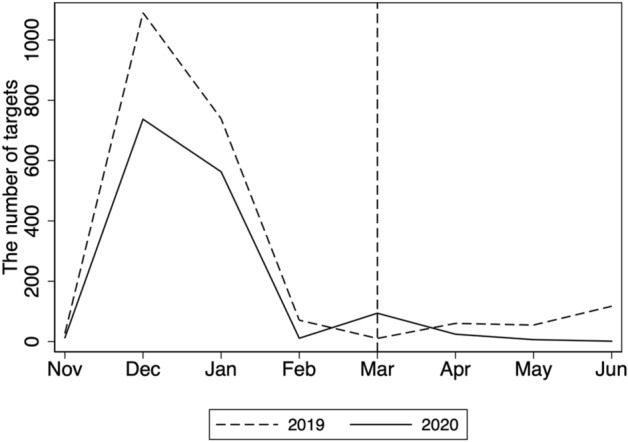
Difference in weekly average number of targets based on complete date. The unit of observation is school. The date of target is defined by the day when a student finishes the target set by a teacher. We exclude students who did not complete the assigned target

**Fig. 27:**
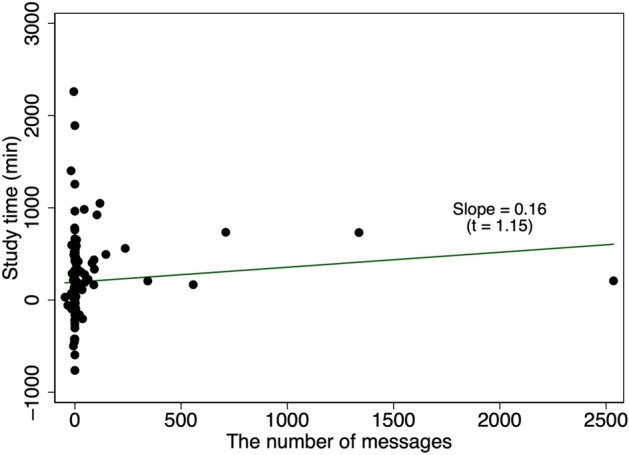
Correlation between study time and messages. The number of observations is 127. The unit of observation is school. Study time represents average study time within each school in March, April, and May. The number of messages represents the total number of messages sent from teachers to students in March, April, and May

**Fig. 28:**
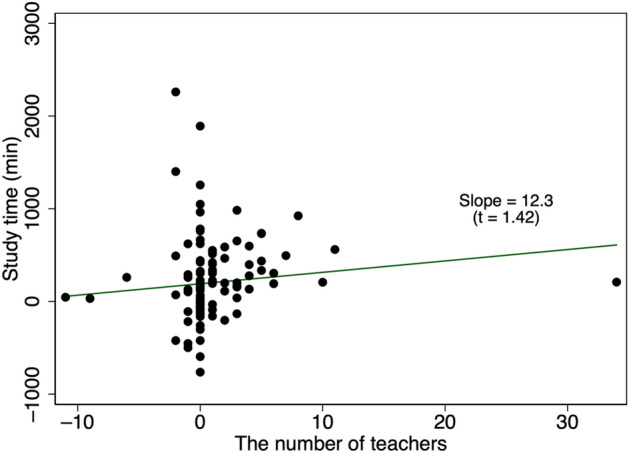
Correlation between study time and the number of teachers online. The number of observations is 127. The unit of observation is school. Study time represents average study time within each school in March, April, and May. The number of teachers online represents the number of teachers who use at least once in March, April, and May

**Fig. 29:**
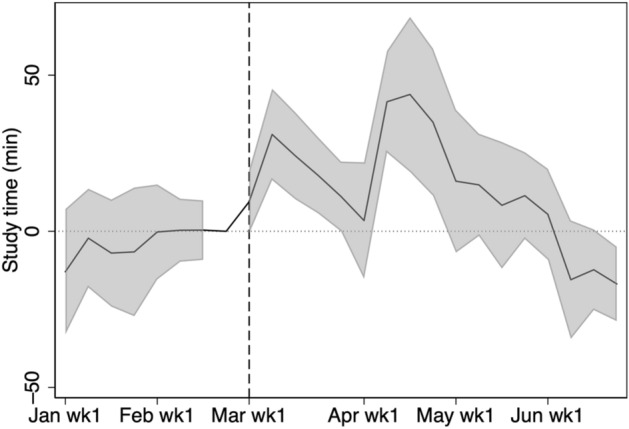
Change of weekly study time from 2019 to 2020 for Jr. HS. The shaded region is the 95% confidence interval computed with standard errors robust to clustering at school level. Study time for students in grades 7 and 8

**Fig. 30:**
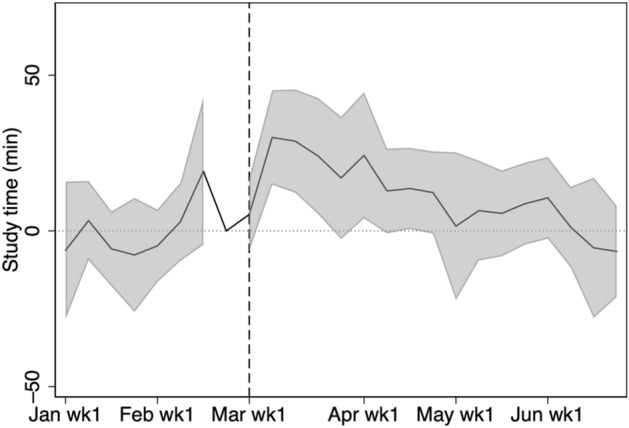
Change of weekly study time from 2019 to 2020 for HS. The shaded region is the 95% confidence interval computed with standard errors robust to clustering at school level. Study time for students in grades 10 and 11

**Fig. 31:**
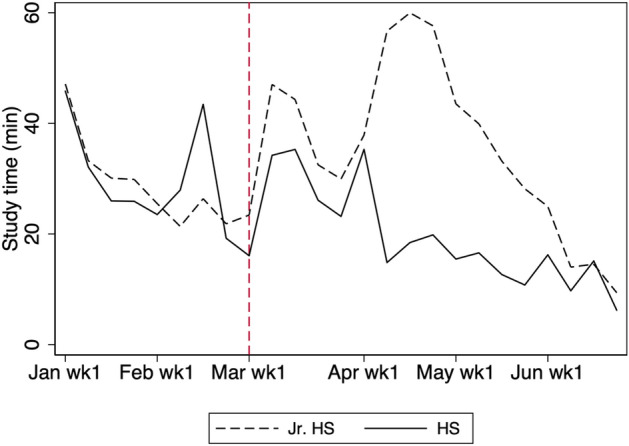
Average weekly study time by grade in 2020. Study time for students in grades 7, 8, 10, and 11 in 2020

**Fig. 32:**
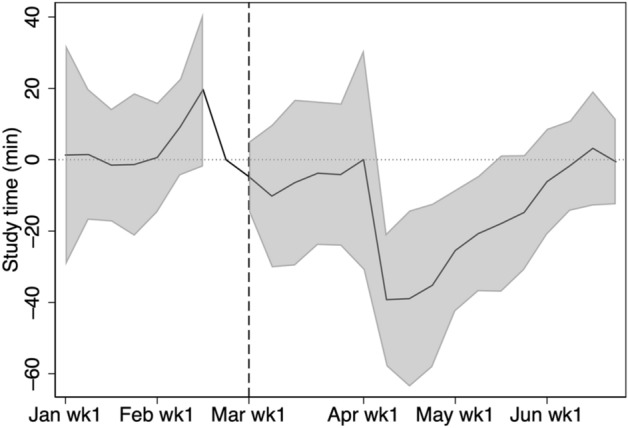
Difference in the growth of study time between Jr. HS and HS students. The shaded region is the 95% confidence interval computed with standard errors robust to clustering at school level. Study time for students in grades 10 and 11 in 2020 minus study time for students in grades 7 and 8 in 2020

**Fig. 33:**
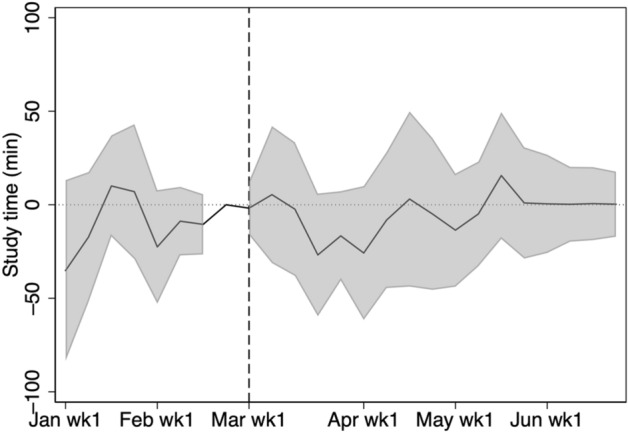
Heterogeneity by school quality for Jr. HS. The shaded region is the 95% confidence interval computed with standard errors robust to clustering at school level. Study time for students in grades 7 and 8 in high-quality school minus study time for students in grades 7 and 8 in low-quality school. School quality is defined by the level of school obtained from external sources

**Fig.34:**
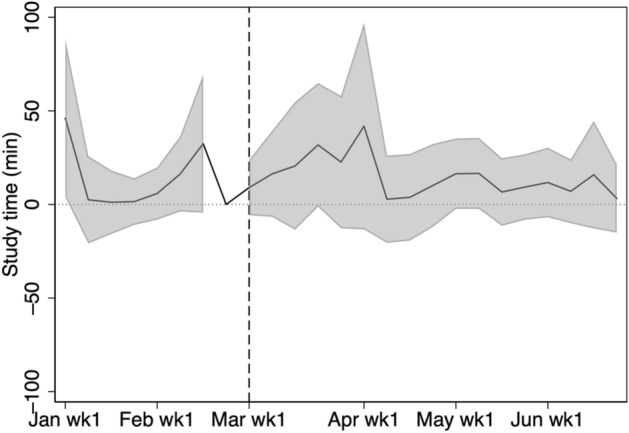
Heterogeneity by school quality for HS. The shaded region is the 95% confidence interval computed with standard errors robust to clustering at school level. Study time for students in grades 10 and 11 in high-quality school minus study time for students in grades 10 and 11 in low-quality school. School quality is defined by the level of school obtained from external sources

**Fig. 35:**
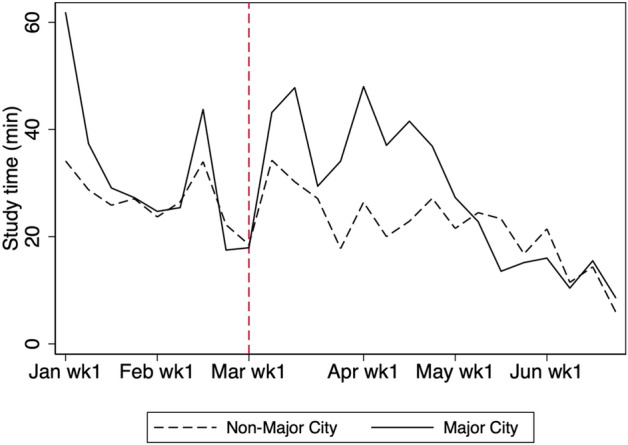
Average weekly study time by region in 2020. Study time by region for students in grades 7, 8, 10, and 11 in 2020. Four cities in Japan, Tokyo, Osaka, Aichi, and Kanagawa, are defined to be major cities

**Fig. 36:**
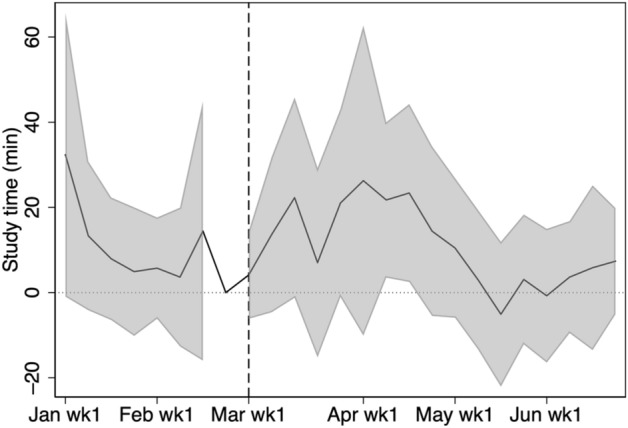
Heterogeneity by region. The shaded region is the 95% confidence interval computed with standard errors robust to clustering at school level. Study time for students in grades 7, 8, 10, and 11 in a school located in a major city minus study time for students in grades 7, 8, 10, and 11 in a school located in a non-major city. Four cities in Japan, Tokyo, Osaka, Aichi, and Kanagawa are defined to be major cities

**Fig. 37:**
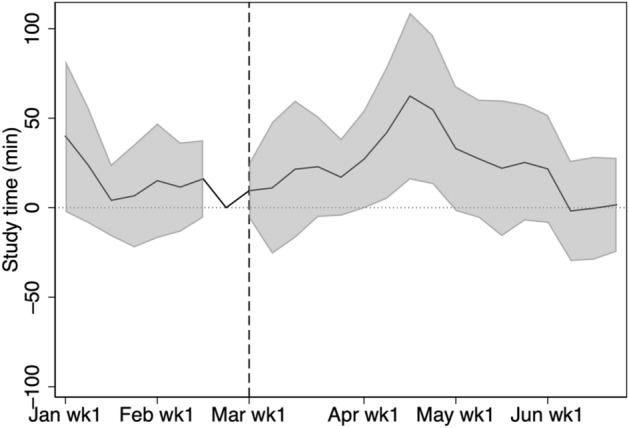
Heterogeneity by the previous usage for Jr. HS. The shaded region is the 95% confidence interval computed with standard errors robust to clustering at school level. Study time for students in grades 7 and 8 in a school with high usage minus study time for students in grades 7 and 8 in a school with low usage. The previous usage is defined by the average study time for students in a given school from April 2019 to December 2019. Low and High are defined by a student belonging to a school with the previous usage below median and above median, respectively

**Fig. 38:**
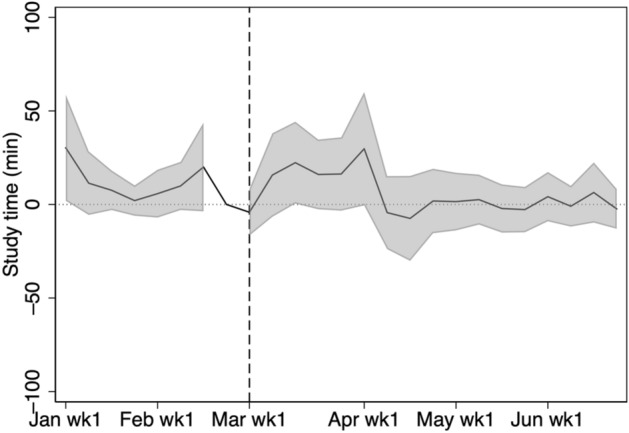
Heterogeneity by the previous usage for HS. The shaded region is the 95% confidence interval computed with standard errors robust to clustering at school level. Study time for students in grades 10 and 11 in a school with high usage minus study time for students in grades 10 and 11 in a school with low usage. The previous usage is defined by the average study time for students in a given school from April 2019 to December 2019. Low and High are defined by a student belonging to a school with the previous usage below median and above median, respectively

**Fig. 39:**
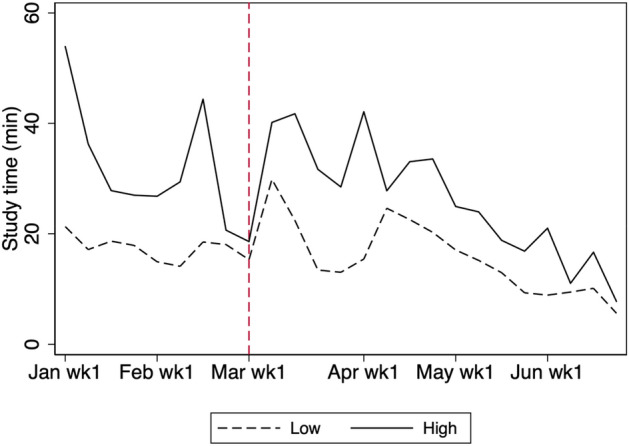
Average weekly study time by the previous usage in 2020. Study time by the previous usage for students in grades 7, 8, 10, and 11 in 2020. The previous usage is defined by the average study time for students in a given school from April 2019 to December 2019. Low and High are defined by a student belonging to a school with the previous usage below median and above median, respectively

**Fig. 40:**
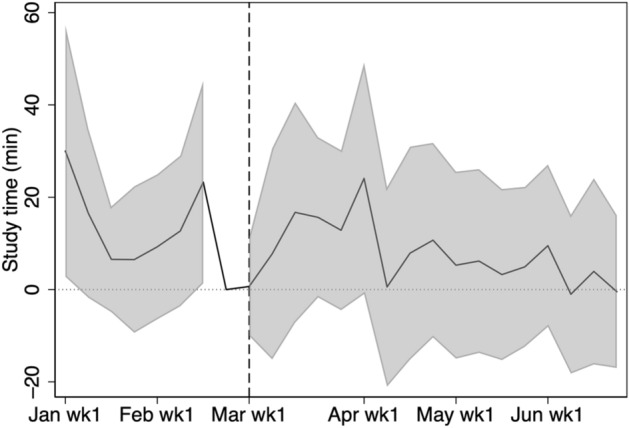
Heterogeneity by the previous usage. The shaded region is the 95% confidence interval computed with standard errors robust to clustering at school level. Study time for students in grades 7, 8, 10, and 11 in a school with high usage minus study time for students in grades 7, 8, 10, and 11 in a school with low usage. The previous usage is defined by the average study time for students in a given school from April 2019 to December 2019. Low and High are defined by a student belonging to a school with the previous usage below median and above median, respectively

**Fig. 41:**
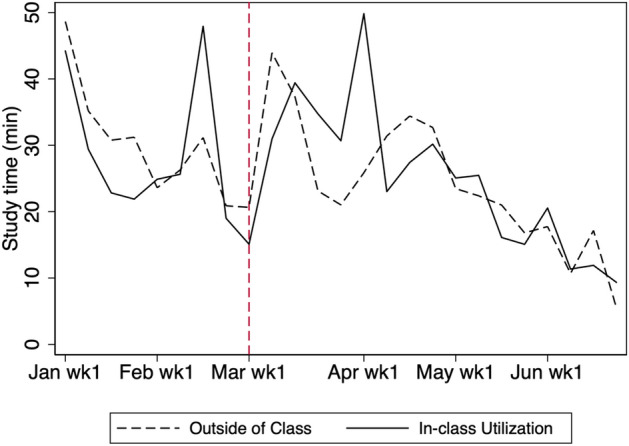
Average weekly study time by in-class utilization in 2020. Study time by whether the platform is used in class for students in grades 7, 8, 10, and 11 in 2020

**Fig. 42:**
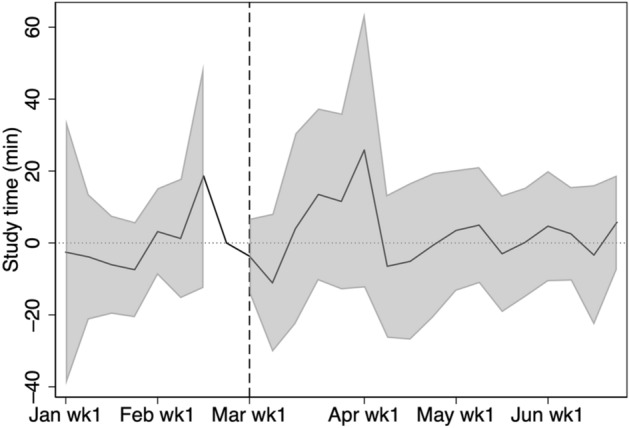
Heterogeneity by in-class utilization. The shaded region is the 95% confidence interval computed with standard errors robust to clustering at school level. Study time for students in grades 7, 8, 10, and 11 in a school using the platform in class minus study time for students in grades 7, 8, 10, and 11 in a school using the platform outside class

## Appendix I

In this Appendix, we describe additional institutional information: more detailed description of the online learning service on which we focus in this paper, and additional comment on Japanese academic calendar.

First, Surala is run by a private company, SuRaLa Net Co.,Ltd., which is independent of any public entities. The company start off, primarily focusing on the business-to-business market, in 2007. In 2012, the company extend its business to the business-to-consumer market.

The service is adopted by various entities in different grades such as high school, elementary school, individuals, and private tutors. In our analysis, we mainly focus on school-level utilization of the service in junior high school and high school. In the case where a school makes a contract with the company, the school ask the company to issue accounts for student. Then, school distributes those accounts to the students and instructs them the way to study in this service. Students can freely login the service any time after distribution of their account. All the materials in subjects under the contract is available to the students.

The service offers study materials in five subjects: Math, English, Japanese, science, and social study. Some subjects are not available to certain grades. For instance, there is no high school-level science and social study materials available.

In the service, three types of functions are available: lectures, drills, and tests. In the lecture part, students watch recorded video and answer brief quizzes prepared by a teacher during the lecture. The drills are offered not only in the form of multiple-choice form as in brief quizzes in the lecture but also in various different ways such as open-ended questions and dictation. Finally, difficulty of the drills is adjusted depending on the level of individual ability.

The platform offers three types of tests. The first test is a general assessment of their academic ability. The second test is a mock exam for their mid-term and final exams. The third test is a short quiz taking around ten minutes. In the second and third test, the scope of topics in the test can be specified so that students can selectively study certain topics.

As of March in 2018, 151 schools, most of which is a private school, are under contract. The price of the service is around 7500–10,000 yen (roughly 71–95 USD) per month in individual case depending on the number of subjects and the type of contracts. Although this price should be higher than the price for school-level contract, the budget for technological advancement in public school, for instance less than 10,000 yen (roughly 95 USD) per year in the case of governmental policy in 2014, cannot afford adoption of the service. Furthermore, before COVID-19, Japanese educational system did not allow elementary school and junior high school to incorporate a class solely based on online materials into their official curriculum. In high school, only the limited number of classes based on online materials can be incorporated into their official curriculum.

School uses the service in and out of class. Some school, for example, can use this platform to teach English in class while other school can use it as homework for Math class. We have data on the ways of their usage and the purpose before and after school closure. However, even within schools categorized as the same way of usage in this data, there are quite a little variation in their actual study time. Thus, we do not clearly observe the purpose of usage in each school.

Second, even though nationwide school closure due to COVID-19 began from March 2 and ended in the end of May, the effective number of lost school days is less than 3 months. This is because there are three events in which schools would not have classes even in the absence of school closure: spring break, transition of academic year, and national holidays.

First, spring break begins typically from the third or fourth week of March and lasts in the first week of April. Second, most schools do not conduct classes for ceremonial and administrative procedures at the end and beginning of an academic year. Finally, in the beginning of May, there are three consecutive national holidays, from Monday to Wednesday. This means that the number of classes hold in this week is less than other weeks. Combining all of these events, roughly 8–9 weeks of classes have not taken place in school due to COVID-19.

## Appendix II

The service provider SuRaLa Net Co., Ltd. conducted a survey for schools using Surala to see how schools changed the ways they use the service during school closure. The survey was responded by a teacher representing a school.

Figure 18 presents the purposes of using the service. Schools chose one of the following three alternatives: preparation, review, and compensatory education. In pre-COVID-19 period, most (77%) schools used Surala for review, meaning that students study materials that have recently been taught in class. The share of review drops to 47% during school closure, but the share of schools that use Surala for preparation for new materials rose from 4% to 40%. This shift from review to preparation suggests that schools tried to make up for lost class using Surala during school closure. Last, the share of compensatory education, meaning that students study materials taught in a lower grade, slightly increased from 13% to 20%.

Figure 19 shows when students use the service in the day. Before school closure, students are almost equally likely to use the service in either class, after class, or at home. During school closure, students use Surala either in class, in time assigned by teachers, or any time in the day. Vast of majority (89%) of schools did not specify the time when students study using Surala. Their students are free to choose when to use Surala in the day during school closure.

Finally, Figure 20 presents how students use the service. In most schools, teachers assign units to students both before and during school closure. The share of schools that use Surala for quizzes and review, meaning that schools instruct students first to take a test and to review the mistakes, slightly increased from 4 to 12%. Overall, we do not find significant change in these dimensions of usage between before and during school closure.

## Appendix III

In this appendix, we discuss another variable, which may capture teachers’ input. In the online learning service, teacher can set target for students. The company provide the information of when the target is set, when the target is supposed to be finished, and when the target is actually finished.

Overall, we find target is mostly used in the beginning of winter break, December, and the beginning of academic calendar, April. Even though we observe slight increase after school closure, the amount of increase is quite small comparing to the beginning of winter break and the beginning of academic calendar. Thus, we find it difficult to detect the effect of school closure on this variable except we observe almost no target usage in the beginning of academic calendar in 2020 (Figs. 21, 22, 23, 24, 25, 26).

## Appendix IV

In Sect. 2, we discuss correlation between study time and messaging with the graphs without outliers. In this Appendix, we present the graphs with full sample (Figs. 27, 28).

## Appendix V

In this Appendix, we report heterogeneity analyses concerning variables not presented in Sect. 5. To be more specific, we present heterogeneous effects by grade, by school quality, by region, by previous usage, and by in-class utilization.

### By grade

We examine heterogeneity of the responses to school closure by comparing the growth of study time from 2019 to 2020 between groups. There are a few reasons why responses may be different between Jr. high school and high school. First, high school and junior high school are different in contents and intensity of study. Second, we expect different selection into our sample. For instance, students enter public junior high school unless they choose to take entrance exam for private junior high school while both private and public high school require entrance exams. Finally, a manager of the company mentioned that students in the junior high schools in our sample tended to have better background than students in the high school. This difference may be particularly salient during the school closure because students cannot use the service without their own computer at home when their schools are closed.

Figure ^29^ shows the change of study time for Jr. high school students, while Fig. ^30^ shows that for high school students. For both Jr. high school and high school students, the growth of study time is significant in March and April, although the magnitude is greater for Jr. high school students. We also directly compare the study time in 2020 between groups in Figs. ^31^ and ^32^. In April and May, Jr. high school students increase their study time significantly more than high school students do.

A possible explanation behind this heterogeneity is differences in students’ background. Japanese compulsory education covers Jr. high school, but not high school. Majority attends public Jr. high school, but students from relatively wealthy families tend to go to private Jr. high school. By contrast, private institutions are common among high schools and not necessarily for wealthier students. Although we do not observe the wealth of students’ families, we expect that Jr. high school students in our sample have better family socioeconomic status than high school students in our sample.

### By school quality

In Figs. 33 and 34, we find different pattern for high school and junior high school even though the pattern is not statistically significant. Only heterogeneity across school quality in high school in March is marginally statistically significant.

### By region

Figures 35 and 36 shows regional difference in average weekly study time. In this analysis, we define four cities (Tokyo, Aichi, Osaka, Kanagawa) as major cities. The fraction of students in major cities is around 46%. In major cities, we observe a marginally statistically significant difference between two categories. From March to April, students in major city tend to study longer than the other group.

### By the previous usage

We examine heterogeneity with respect to the past utilization, specifically past study time and in-class utilization before school closure. We expect that different experience before COVID-19 may affect utilization during school closure. For instance, schools with large amount of past utilization may be affected by school closure more intensely because they are more familiar with the service. On the other hand, they may have little room for further increase in the amount of utilization.

Figures 37, 38, 39, and 40 describe heterogeneity with respect to the past study time. We define utilization category based on the school-level average study time from April 1st to December 31st in 2019. The fraction of students who belong to school with high utilization is around 83%. Overall, schools with higher past utilization consistently study longer in the service after COVID-19, as well as before COVID-19. As in Fig. 40, however, there is no statistically significant difference between two groups. Figures 37 and 38 show heterogeneity in high school and junior high school separately. In high school, there is a marginally statistically significant effect in March while, in junior high school, there is a statistically significant effect in April. Both of the patterns observed here is consistent with the pattern observed in the analysis of the heterogeneity across grade. For instance, the effect in high school is strongest in March.

### By in-class utilization

Figures ^41^ and 42 shows the difference in average weekly study time across the way school utilize the platform before school closure. In-class utilization of the platform before school closure is defined based on the information obtained through a survey. The fraction of students in a school with in-class utilization is around 43%. There is no statistically significant difference between two groups.

**Table 1:** Rates of school closure

	April 22 (%)	May 11 (%)	June 1 (%)
Public schools			
Junior high schools	95	88	1
High schools	99	92	8
Private schools			
Junior high schools	97	90	8
High schools	98	88	6

The table shows the percentage of schools that were open each day on the dates specified

Source: https://www.mext.go.jp/a_menu/coronavirus/mext_00007.html

**Table 2:** Descriptive statistics

	Observations	Mean	SD	Median	Min	Max
Time spent for one unit (min)	1,722,701	8.98	11.37	5.35	0.02	141.85
Indicator for log-in in a given week	624,175	0.32	0.47	0.00	0.00	1.00
Weekly study time conditional on log-in (min)	192,469	73.84	93.15	40.95	0.05	814.75
Weekly unconditional study time (min)	610,225	23.29	62.56	0.00	0.00	814.75
Indicator for high school	21,454	0.68	0.47	1.00	0.00	1.00
Indicator for male	21,454	0.55	0.50	1.00	0.00	1.00
No access from home	16,424	0.17	0.38	0.00	0.00	1.00
Number of students per school	224	95.78	160.45	22.50	1.00	1032.00
Average study time Apr–Dec 2019 (min)	164	596.67	681.36	373.46	11.57	4121.09
School quality index for junior high school	51	40.30	6.85	39.00	31.00	59.60
School quality index for high school	95	49.81	7.02	50.50	35.50	67.00
Indicator for messages sent in 2020	4,596	0.95	0.22	1.00	0.00	1.00
Weekly number of messages at the school level	431	10.66	27.47	3.00	1.00	264.00
Weekly number of teachers online at the school level	431	1.41	1.00	1.00	1.00	11.00
Weekly number of messages at the teacher level	608	7.56	13.98	2.00	1.00	104.00

The time spent for one unit is defined by the average study time per unit. Indicator for log-in in a given week is defined by the dummy variable indicating whether a student studies at least once a week. We use the school-level indicator obtained from external sources as a school quality index. The average study time April–December 2019 is defined by the average study time per student in each school in April–December 2019. The weekly number of messages at the school level is defined by the number of messages that teachers in a given school send in a given week. The weekly number of teachers at the school level is defined by the number of teachers who use messages in a given week. The weekly number of messages at teacher level is defined by the total number of messages sent by one teacher in a given week

**Table 3:** Monthly-level analyses of study records

	January	February	March	April	May	June
Panel A. Unconditional						
2019	37.93	21.792	10.595	14.627	12.963	20.142
	[3.462]	[2.054]	[1.744]	[2.052]	[1.681]	[3.350]
2020	31.431	25.618	32.386	30.102	19.435	11.057
	[2.674]	[2.890]	[3.999]	[3.743]	[2.782]	[1.937]
Difference	− 6.499***	3.827	21.791***	15.476***	6.472***	− 9.085***
	[2.967]	[3.237]	[3.827]	[3.317]	[2.949]	[3.345]
Panel B. Log-in Rate						
2019	–	0.756	0.384	0.476	0.433	0.457
	–	[0.032]	[0.048]	[0.047]	[0.044]	[0.054]
2020	–	0.802	0.51	0.514	0.362	0.327
	–	[0.028]	[0.046]	[0.050]	[0.042]	[0.046]
Difference	–	0.046	0.126***	0.038	− 0.071	− 0.131***
	–	[0.033]	[0.052]	[0.048]	[0.048]	[0.050]
Panel C. Conditional						
2019	37.936	28.836	27.598	30.752	29.971	44.039
	[3.463]	[2.340]	[3.635]	[2.754]	[2.855]	[3.989]
2020	31.438	31.954	63.461	58.632	53.771	33.846
	[2.675]	[3.334]	[5.289]	[5.432]	[4.503]	[4.142]
Difference	− 6.498***	3.118	35.863***	27.881***	23.801***	− 10.193*
	[2.968]	[3.844]	[5.064]	[5.344]	[4.981]	[6.077]

Unconditional represents the study time for students in grades 7, 8, 10, and 11. Conditional represents the study time conditional on log-in for students in grades 7, 8, 10, and 11. Monthly log-in is defined as studying at least once in a given month. Study time is defined as the weekly average study time within each month. The log-in rate for January is blank because the estimation sample is conditioned on the log-in in January. The symbols, *, **, and ***denote significance at the 10%, 5%, and 1% levels, respectively. Standard errors are clustered at school level

Source: Author calculations

**Table 4:** Monthly-level analyses of message data

	January	February	March	April	May	June
Panel A Number of Messages						
2019	0.041	0.035	0.037	0.04	0.11	0.453
	[0.018]	[0.013]	[0.019]	[0.017]	[0.036]	[0.200]
2020	0.092	0.059	0.288	1.83	1.456	0.278
	[0.024]	[0.018]	[0.075]	[0.868]	[0.485]	[0.112]
Difference	0.052*	0.024	0.252***	1.79**	1.346**	–0.175
	[0.030]	[0.022]	[0.077]	[0.868]	[0.487]	[0.229]
Panel B Number of Teacher Online						
2019	0.01	0.02	0.01	0.013	0.034	0.034
	[0.004]	[0.005]	[0.004]	[0.005]	[0.008]	[0.008]
2020	0.029	0.026	0.045	0.096	0.133	0.043
	[0.006]	[0.007]	[0.008]	[0.026]	[0.030]	[0.015]
Difference	0.019**	0.006	0.035***	0.084**	0.099**	0.009
	[0.007]	[0.008]	[0.009]	[0.027]	[0.031]	[0.017]
Panel C Messages Per Teacher						
2019	3.417	2.125	3.375	4.5	3.644	7.423
	[0.935]	[0.713]	[1.256]	[1.645]	[0.788]	[4.604]
2020	2.414	2.04	5.476	13.899	9.538	4.115
	[0.394]	[0.236]	[1.192]	[7.219]	[3.507]	[1.659]
Difference	–1.002	–0.085	2.101	9.399	5.893	–3.308
	[0.930]	[0.665]	[1.624]	[7.378]	[3.553]	[5.405]

Number of messages indicates the weekly average number of messages from teachers to students per school. Per school number of teachers online represents the number of teachers who send at least one message in a given period. Messages per teacher represent the average number of messages per teacher conditional on being online. All of these three measures are weekly averages within each month. The symbols, *, **, and ***denote significant at the 10%, 5%, and 1% levels, respectively. In Panel C, standard errors are clustered at school level.

Source: Author calculations

## Footnotes

1 The specific number cited is from https://en.unesco.org/covid19/educationresponse.

2 More detailed information can be obtained from https://apts.org/news/station-stories/public-media-education-partnerships-school-districts-governments-and-education-agencies.

3 As of April 28, 2021, only 4 articles about educational consequences of COVID-19 are published in COVID ECONOMICS by the CERP, while 68, 53, and 46 articles about “Effects of Containment Measures on Covid-19 contagion”, “Effects of the Virus and Social Distancing on the Economy”, and “Effects on Jobs, Earnings and Household Behaviour” are published. The number of articles is calculated in https://cepr.org/content/covid-economics-searchable-index, except that the number of articles about education is calculated by the authors because there is no categorization tag indicating articles about education.

4 The information in this section is obtained from the following three websites: https://www.kantei.go.jp/jp/singi/novel_coronavirus/taisaku_honbu.htmlhttps://kokkai.ndl.go.jp/#/ https://www.pref.tottori.lg.jp/item/1204674.htm.

5 The actual administrative term used by the government is prefecture, but we use province as it is more intuitive to most readers.

6 The data were downloaded from minkou.jp in June 2020. A marketing research company claims that this website is the most viewed of those providing information on schools in Japan and we confirmed during informal interviews that teachers, students, and their parents often referred to the website. Although other firms publish high school quality data, the data are very similar between firms.
